# Eco-Friendly Plant-Derived Fillers (Ginseng, Lemongrass, Turmeric, Wood Flour) for Elastomeric Composites Containing Natural and Chloroprene Rubbers (NR/CR)

**DOI:** 10.3390/polym17243317

**Published:** 2025-12-16

**Authors:** Aleksandra Smejda-Krzewicka

**Affiliations:** Institute of Polymer and Dye Technology, Faculty of Chemistry, Lodz University of Technology, Stefanowskiego 16, 90-537 Lodz, Poland; aleksandra.smejda-krzewicka@p.lodz.pl

**Keywords:** ginseng—*Panax ginseng*, lemongrass—*Cymbopogon citratus*, turmeric—*Curcuma longa*, wood flour, biofiller, chloroprene rubber, natural rubber

## Abstract

This work aimed to investigate the properties of cross-linked elastomeric blends based on natural rubber (NR) and chloroprene rubber (CR), incorporating plant-derived fillers as environmentally friendly additives. The selected eco-friendly biofillers included ginseng, lemongrass, turmeric, or wood flour. In situ surface modification with *n*-octadecyltrimethoxysilane was carried out to enhance the compatibility between the fillers and the elastomeric matrix. The results showed that both unmodified and silane-modified plant-based fillers can be effectively used in NR/CR composites, yielding vulcanizates with favorable performance characteristics. The ginseng-filled composite exhibited the highest degree of cross-linking and superior mechanical strength among the tested materials. Turmeric, in both its unmodified and silane-treated forms, contributed to the greatest resistance against aging factors. Notably, the silane-modified wood flour filler significantly improved tear resistance, nearly doubling that of the unfilled rubber. Overall, these novel rubber composites demonstrate not only promising functional properties but also considerable ecological and economic advantages.

## 1. Introduction

In the ever-evolving field of infrastructure and industry, the role of natural rubber (NR) has become increasingly crucial. Natural rubber is obtained from the Hevea brasiliensis tree. It is recognized as a green and renewable elastomer, primarily consisting of cis-1,4-polyisoprene, along with non-rubber components, including proteins, lipids, and inorganic salts [1,2,3,4,5]. This unique elastomer is the reference standard for all synthetic rubbers. The most important advantage of NR, acquired after its cross-linking, is high tensile strength [6]. Other strengths of cross-linked NR are excellent elastic properties, which result in very low hysteresis, i.e., significant damping of vibrations. This property, combined with the low heat release into the environment during dynamic loads, makes NR an ideal material for producing elements highly exposed to vibrations. Additionally, natural rubber is an insulator of electric current and is resistant to chemical agents such as alcohols, esters, glycols, and ketones.

The primary source of natural rubber, the Hevea brasiliensis tree, provides us with an organic polymer rich in tensile strength, vibration-dampening properties, and great resistance to tear, features that prove indispensable in sectors such as construction and automobile manufacturing. Thriving automobile markets worldwide drive up demand for this valuable material, as do rising requirements for latex products such as gloves, belts, and catheters. The demand for natural rubber is increasing due to its characteristic properties, such as adhesion to metals and resistance to abrasion, which make it suitable for manufacturing seals, tires, and other products. The properties of natural rubber, such as high tensile strength, vibration dampening, and tear resistance, make it ideal and more preferred than synthetic rubber for its application in the automobile industry and large constructions. The largest end-user of rubber is the automotive parts industry, which uses a massive amount of rubber to make pipes, gaskets, car tires, hoses, and other parts every year. The technical disadvantages of cis-1,4-polyisoprene include its poor abrasion resistance compared to other materials. Moreover, NR is not very resistant to fire, weather conditions, and non-polar solvents. When in direct contact with a flame, it burns like most organic substances. NR is also readily oxidized when exposed to heat, oxygen, ozone, or light, with poor resistance to oil and heat [7,8,9]. Natural rubber is also susceptible to degradation by oxidative aging and ozone attack, and it is non-resistant to oil, unlike some polar elastomers [10]. To sum up, the characteristics of natural rubber make it an irreplaceable raw material in many cases. However, it should be remembered that it also has limitations, which may exclude its use in a given system.

The disadvantages of natural rubber can be overcome by combining it with other elastomers, e.g., chloroprene rubber (CR). CR is a vital rubber produced by emulsion polymerization of 2-chloro-1.3-butadiene [11,12]. The dominant elements are 1.4-trans addition units (87%) in the CR macromolecules produced at 40 °C. The remaining fragments of the CR macromolecule are 1.4-cis addition units (10%), −1.2-vinyl addition units (approx. 2%), and −3.4 addition units (approx. 1%) [13]. In the CR structure, there is a small number of units containing a highly active allyl-bonded chlorine atom (mers with −1.2 addition). The presence of units with this structure enables cross-linking CR with metal oxides. Chloroprene rubber is a specialized elastomer, characterized by good mechanical properties, strong adhesion, resistance to technical media, crystallization ability, and thermo-cross-linking [14,15,16]. Increased flame resistance is the most important parameter that characterizes CR [17,18,19]. Chloroprene rubber is standardly cross-linked with zinc oxide (5 phr) in the presence of magnesium oxide (4 phr) [13]. Cross-linking of CR with iron(III) oxide [20], copper(I) oxide or copper(II) oxide [21], or silver(I) oxide [22] is also known. Vulcanization in the presence of metal oxides is expected to reduce the number of residual allylic chlorines in the structure. This is crucial to prevent the release of corrosive gases, such as chlorine or hydrogen chloride [23]. CR macromolecules are characterized by a high degree of regularity in structure and polarity resulting from the inductive effect of the chlorine atom [12,24].

Due to its specific properties, chloroprene rubber is used in many industries, and it is often a component of elastomeric blends. There are many types of rubber blends available in the market. Elastomeric blends are of great importance in polymer processing because they are characterized by high-performance parameters, durability, well-balanced mechanical properties, and a relatively low cost [25,26]. Therefore, rubber blends have gained considerable attention in various applications in all fields of industry and technology [27]. Properly composed combinations of two or more polymers allow you to use the strengths of each component while eliminating the disadvantages. However, such a process involves dealing with incompatibility resulting from the different natures of both elastomers [28,29,30,31].

According to Sae-oui et al. [32], CR/NR composites lead to products with good mechanical properties because both elastomers are strain-induced crystallizable. Adding chloroprene rubber to natural rubber helps overcome natural rubber’s main shortcomings, i.e., poor oil and aging resistance. Meanwhile, the presence of NR in CR compounds improves elasticity and low-temperature flexibility. According to Manohar et al. [33], NR and CR are blended to obtain products with intermediate properties and to reduce the cost (by adding a cheap NR to an expensive CR). Therefore, it is very important to quantify the mass ratio of these elastomers in the cured blends that are required to obtain an optimum balance of desired properties. In the case of chloroprene rubber with a natural rubber blend (prepared at a 50/50 blend ratio), ethylene thiourea as an accelerator can be partially replaced by an alkanolamide [31]. It was found that the use of alkanolamide in the NR/CR composites filled with carbon black exhibited shorter scorch and cure time, higher torque increment, higher elongation at break, and higher tensile modulus (but only with tiny amounts of alkanolamide). No significant differences were observed in other properties, especially tensile strength. It is worth adding that ethylene thiourea was not eliminated in the tested materials.

Sae-oui et al. [32] investigated the properties of the CR/NR composites with a blend ratio of 75/25, filled with different amounts of precipitated silica. It was found that increasing silica loading positively affected the cure characteristics of the blend, and the tensile strength improved slightly with increasing silica loading up to 30 phr. This improvement was attributed to particles and the overall increased cross-link density. However, in the publication [34], CR/NR blends with different blend ratios and filled with a constant amount of silica (40 phr) were tested. It was found that resistance to thermal aging, oil, and ozone increased with increasing CR content because the reaction between allylic chloride atoms of CR and the silanol groups on the silica surface gave additional cross-links and the highest state of cure and a high degree of filler dispersion. It was also found that at a 50/50 CR/NR ratio, the silica-filled CR/NR blend possessed good mechanical properties and satisfactory aging and oil resistance. In the paper [35], silica was grown in situ into an NR/CR blend (at a 40/60 ratio), by a solution sol–gel method, where silica content in the rubber blend was increased in a controlled manner exceeding the limit found for the same blend ratio in the soaking sol–gel method. Tetraethoxysilane was used as a modifier of silica. However, the silica dispersion and the rubber-filler interactions improved drastically by using silane compared to unsilanized silica-filled composite at the same silica content.

CR/NR blends filled with magnesium carbonate (MgCO_3_) are also known [36]. The results revealed that, regardless of the blend ratio (CR/NR: 75/25, 50/50, 25/75), increasing MgCO_3_ loading resulted in not only an increase in the compound viscosity but also enhancements of the cure rate and state of cure. For CR/NR blends, a plateau behavior was obtained at a 50/50 blend ratio, where the degrees of post-curing and reversion were counterbalanced. The results also reveal that when MgCO_3_ was loaded at low concentrations (≤20 phr), the degree of filler–filler interaction was very low and had no significant effect on the compound properties. However, the publication does not present the functional properties of the produced vulcanizates, so it is not easy to assess the effect of MgCO_3_ on the final properties of the CR/NR composite, and this aspect is the most important for the practical use of new materials. The functional properties of CR/NR vulcanizates are presented in the publication [37]. A chloroprene and natural rubber blend filled with a multi-walled carbon nanotube (MWCNT) was tested. The effects of the blending process on the morphology of the conductive network, interface interaction, and resistance–strain response behavior of MWCNT/CR–NR composites were systematically studied. Compared with pure CR and NR, CR/NR blends have better tensile strength and elongation at break, and their moduli are between those of pure CR and pure NR. Previous research has proved that CR blends with NR are incompatible, so Fulin et al. [38] applied CR/NR blends filled with kaolin processing aid Struktol WB212 (a mix of fatty acid ester with high molecular weight and medium activated filler) to prevent CR compound sticking to rolls in early stage mixing, and homogenizing agent Struktol 60NSF (a mix of aromatic, cyclone, and aliphatic hydrocarbons) to disperse the rubber components.

Compositions containing natural rubber and chloroprene rubber are not common rubber materials due to differences in their polarity. Therefore, they are most often manufactured conventionally, i.e., cross-linked with standard curing agents and filled with active fillers. Plant-derived fillers are not often used for this type of composition. Among the biofillers most commonly used in elastomer technology is readily available cellulose (obtained from trees, grasses, leaves), biodegradable, and inexpensive. Its fibers can be used as rubber modifiers to improve elasticity and mechanical properties. For example, Datta et al. [39] studied the effect of cellulose on the properties of natural rubber and found that all cellulose-filled vulcanizates had poorer abrasion resistance than unfilled materials, and cellulose content above 10 phr led to reduced elasticity, tensile strength, and deformation resistance. The optimum amount of cellulose in the elastomeric matrix is in the range of 10 phr to 15 phr [40,41,42]. Nonetheless, other studies have shown that adding cellulose to an elastomeric matrix results in an increase in thermal stability, crystallization temperature, and mechanical strength [43,44,45]. According to [46] oak bark can be used as a biofiller in natural rubber composites. The oak bark modified with n-octadecyltrimethoxysilane exhibited enhanced dispersion and reduced aggregates in the elastomeric matrix. The incorporation of oak bark improved aging resistance at least two-fold due to phenolic derivatives with antioxidant properties. Hydrophobicity decreased with added bark, but silanization reversed the trend, making samples with a high content of oak bark the most hydrophobic.

Starch, obtained from corn, wheat, potatoes, cassava, or tapioca, can also be used as a biofiller for elastomers. Chemically, starch is a polysaccharide containing two main fractions: amylose and amylopectin. The use of starch as a rubber reinforcing filler has been known for a long time [47,48,49,50]. However, the use of starch as a filler also has many disadvantages, such as large particle size, strong polar surface, high cohesion energy, and high melting point. These are among the obstacles to using starch as a filler on a wide scale in the rubber industry. In paper [51], silanized starch was used as an innovative filler for natural rubber composites. Corn starch was chemically modified by silanization to produce a hydrophobic starch with a smaller particle size. The modified starch dispersed better in the natural rubber matrix and obtained a more homogeneous morphology. The higher starch content allowed the composites to achieve a higher degree of cross-linking, resulting in better resistance to swelling in organic solvents. The improved mechanical properties and good dynamic properties, as well as improved hydrophobicity, were mainly due to improved interfacial interactions between rubber and starch.

This literature review highlights the potential and new approaches to using plant-based substances as sustainable biofillers, demonstrating their ability to improve the performance of elastomer composites while supporting more eco-friendly material solutions for the rubber industry. Therefore, this work aimed to investigate the possibility of using ginseng, lemongrass, turmeric, or wood flour as new environmentally friendly bio-fillers of rubber materials. For this purpose, new elastomeric blends containing natural rubber and chloroprene rubber and filled with plant substances were investigated. In this work, a dualistic method of cross-linking NR/CR blends was employed, consisting of sulfur and an accelerator (a typical curing system for NR) and a mixture of zinc oxide, magnesium oxide, and an accelerator (a typical curing system for CR). The mechanical, thermal, and dynamic properties of the filled bio-materials were evaluated, and for comparison, the properties of the unfilled NR/CR vulcanizate were also investigated.

## 2. Materials and Methods

### 2.1. Materials

In this study, natural rubber, NR (RSS from Torimex Chemicals Sp. z o. o., Konstantynów Łódzki, Poland), and chloroprene rubber, CR (DCR-42 from Denka Company Limited, Tokyo, Japan) were used.

The curing system consisted of the following substances:

- sulfur (S) as a cross-linking agent, with a density of 1.8–2.36 g/mL (delivered by Chempur, Piekary Śląskie, Poland),
- zinc oxide (ZnO) as a cross-linking agent, with a density of 5.6 g/mL (delivered by Chempur, Piekary Śląskie, Poland),
- magnesium oxide (MgO) as a cross-linking agent, with a density of 3.6 g/mL (delivered by Chempur, Piekary Śląskie, Poland),
- 1.3-ethylene thiourea (ETU) as a vulcanization accelerator (delivered by Acros Organics, Poznań, Poland),
- N-cyclohexyl-2-benzothiazole sulfonamide (CBS) as a vulcanization accelerator (delivered by Pol-Aura Odczynniki Chemiczne, Morąg, Poland),
- stearic acid (SA) as a cross-linking activator and dispersing agent, with a density of 0.94 g/mL (delivered by Chempur, Piekary Śląskie, Poland).

The following fillers were used:

- ground ginseng, G (*Panax ginseng*) delivered by HerbaNordPol, Gdańsk, Poland,
- ground lemongrass, LG (*Cymbopogon citratus*) delivered by HerbaNordPol, Gdańsk, Poland,
- ground turmeric, T (*Curcuma longa*) delivered by HerbaNordPol, Gdańsk, Poland,
- wood flour, WF (waste raw material), delivered by Scabrosus Sp. z o. o., Wrocław, Poland.

As a selected filler modifier, 95% n-octadecyltrimethoxysilane (C_21_H_46_O_3_Si) with a density of 0.88 g/mL, and 85% n-isomer, supplied by abcr GmbH, Karlsruhe, Germany, was used.

As solvents to study the degree of cross-linking, the following substances were used:

- toluene with a density of 0.87 g/mL, delivered by POCh S.A., Gliwice, Poland,
- diethyl ether with a density of 0.71 g/mL, delivered by Chempur, Piekary Śląskie, Poland.

### 2.2. Research Methods

The recipes for compounding the NR/CR composites are given in Table 1. The elastomeric composites were prepared using a Krupp–Gruson laboratory two-roll mill (Laborwalzwerk 200 × 450, Krupp–Gruson, Magdeburg-Buckau, Germany) with a roll diameter of 200 mm and a length of 450 mm. The temperature of the roll was 20–25 °C, while the speed of the front roll was 20 rpm, with the roll’s friction 1:1.25. The total time to create the filled composition was 10 min. First, the rubbers were plasticized, and the ingredients were incorporated in the following order: stearic acid, ZnO, MgO, filler (with or without silane), accelerators, and sulfur. The obtained rubber composites were stored separately in tightly closed foils at room temperature. The prepared samples were conditioned for 24 h.

Vulcametric measurements were determined by the Alpha Technologies MDR 2000 rotorless rheometer (MDR 2000, Alpha Technologies, Hudson, OH, USA), heated to 160 °C. The oscillation frequency was 1.67 Hz. The test was 30 min and was performed according to ASTM D5289 [52]. The maximum torque increment was calculated from Formula (1):

(1) $${∆T}_{max}=T_{max}-T_{min}$$

where T_max_ is the maximum torque (dNm), and T_min_ is the minimum torque (dNm).

The cure rate index (CRI), a measure of the cross-linking rate, was calculated from Formula (2):

(2) $$CRI=\frac{100}{t_{90}-t_{02}}$$

where t_90_ is the vulcanization time (min), and t_02_ is the scorch time (min).

Vulcanization was performed in an electrically heated hydraulic press. Appropriate amounts of the compositions were placed in the steel molds, which were placed in a press at a temperature of 160 °C and a pressure of 200 bar. The vulcanization time was 5 min. A polyethylene terephthalate foil was used to remove the molded parts from the molds, which were removed after the process. The samples prepared as described above were conditioned for 24 h at room temperature.

The hydrodynamic diameter of the biofiller samples was measured in aqueous dispersions using dynamic light scattering (DLS) measurements. A 1 mg powder sample was weighed, and 20 mL of distilled water was added. The resulting solutions were sonicated for 30 min at 30 °C, and the dispersions were then poured into spectrophotometric measuring cuvettes. Five replicate measurements were performed for each sample using a ZetaSizer Nano-S90 from Malvern Instruments Ltd. (Malvern, Worcestershire, UK).

The surface morphology of the biofillers and cross-section morphology of the vulcanizates were evaluated using a scanning electron microscope (SEM), Hitachi Tabletop Microscope TM-1000 (Tokyo, Japan). The preparation of samples for measurement consisted of placing a double-sided self-adhesive foil on the special tables and gluing the tested sample to it. Then, a gold layer was applied to the prepared sample using the Cressington Sputter Coater 108 auto vacuum sputtering machine (Redding, CA, USA) at a pressure greater than 40 mbar for 60 s. The sample prepared in this way was placed in a scanning electron microscope chamber, and the measurement was performed. Using the TM-1000 software (Ramsay, NJ, USA, 2018), the results were recorded on a computer that cooperated with the spectrophotometer.

The infrared spectra of the biofillers were developed with a Thermo Scientific Nicolet 6700 FT-IR spectrometer equipped with a Smart Orbit ATR (Waltham, MA, USA) diamond attachment, using the attenuated total reflectance (ATR) method. The spectra were assessed for the wavenumber range of 3500–700 cm^−1^. Before the spectra of the samples were collected, background measurements were carried out, each time including 64 scans. Identification of the absorbance bandwidth intensities helped determine the characteristic functional groups present in the structures of the tested biofllers.

The differential scanning calorimetry (DSC), leading to the assessment of the thermal changes and cross-linking temperature range of the elastomeric blends, was investigated using a DSC1 from Mettler Toledo (Mettler-Toledo, Columbus, OH, USA). The thermal properties were measured over a temperature range of −140 °C to 240 °C, at a heating rate of 10 °C/min, and using liquid nitrogen as a coolant. Nitrogen (80 mL/min) was used as a protective gas, whereas liquid nitrogen was applied to cool the sample before the measurement.

The determination of equilibrium volume swelling was performed according to ASTM D471 [53]. Samples were cut from the prepared vulcanizates in four different shapes. Each weighed from 25 mg to 50 mg, with an accuracy of 0.1 mg. Then, the samples were placed in a weighing bottle with solvents, toluene, and hexane. Prepared samples were placed in a thermostatic chamber for 72 h at 25 °C, and after this time were bathed with diethyl ether, dried on filter paper, and then weighed again. Then, the samples were dried in a dryer at 50 °C for 24 h to a constant weight and reweighed. The equilibrium volume swelling (Q_V_) was calculated from Formula (3):

(3) $$Q_{V}=Q_{W}\cdot \frac{d_{v}}{d_{s}}$$

where Q_W_ is the equilibrium weight swelling (mg/mg), d_v_ is the vulcanizate density (g/cm^3^), and d_s_ is solvent density (g/cm^3^).

The equilibrium weight swelling was calculated from Formula (4):

(4) $$Q_{W}=\frac{m_{s}-m_{d}}{m_{d}^{*}}$$

where m_s_ is the swollen sample weight (mg), m_d_ is the dry sample weight (mg), and m_d_* is the reduced sample weight (mg).

The reduced sample weight was calculated from Formula (5):

(5) $$m_{d}^{*}=m_{d}-m_{0}\cdot \frac{m_{m}}{m_{t}}$$

where m_0_ is the initial sample weight (mg), m_m_ is the mineral content in the blend (mg), and m_t_ is the total weight of the blend (mg).

The content of the eluted fraction in toluene (W_Q_) of vulcanizates, interpreted as the amount of leaching substances, was calculated from Formula (6):

(6) $$W_{Q}=\frac{m_{0}-m_{d}^{*}}{m_{0}}$$

The rubber volume fraction (V_r_) was calculated from Formula (7):

(7) $$V_{r}=\frac{1}{1+Q_{V}}$$

The degree of cross-linking (α_c_) was determined by Formula (8):

(8) $$\alpha_{c}=\frac{1}{Q_{V}}$$

Mechanical properties: stress at elongation 100%, 200%, and 300% (S_e100_, S_e200_, and S_e300_), tensile strength (TS_b_), and elongation at break (E_b_) were tested by the universal testing machine ZwickRoell (model 1435, Ulm, Germany). The tests were performed according to PN-ISO 37:2017 [54]. In this method, five samples in the shape of B-type paddles with a measuring section width of 4 mm were used.

The thermo-oxidative aging was tested using the Geer method. The samples to be measured were made in the same way as for the tensile strength test. Five B-type paddles with a measuring section width of 4 mm were cut from each vulcanizate. The prepared samples were placed in a forced-air thermostat at 70 °C. After 7 days, the samples were removed to investigate changes in their strength properties using a ZwickRoell machine (model 1435, Ulm, Germany). Based on the obtained tensile properties, the aging factor (AF) was calculated for the individual vulcanized products by Formula (9):

(9) $$AF=\frac{{{TS}_{b}}^{*}\times {E_{b}}^{*}}{{TS}_{b}\times E_{b}}$$

where TS_b_ is the tensile strength, E_b_ is the elongation at break, TS_b_^*^ is the tensile strength after thermo-oxidative aging, and E_b_^*^ is the elongation at break after thermo-oxidative aging.

Hysteresis losses (∆W_i_) were determined using a testing machine (Zwick1435/Roell GmbH & Co. KG, Ulm, Germany). Each test was performed on three specimens, which were stretched five times to 200% elongation at a tensile speed of 500 mm/min, with an initial force of 0.1 N. The Mullins effect (E_M_) was calculated from the hysteresis loop measurements according to Formula (10):

(10) $$E_{M}=\frac{W_{1}-W_{5}}{W_{1}}\times 100\%$$

where W_1_ is the work required to stretch the sample in the 1st cycle [Nmm], and W_5_ is the work required to stretch the sample in the 5th cycle [Nmm].

Measurements of the Payne effect (∆G′), storage modulus (G′), and loss modulus (G″) were made on the vulcanizate disks with a diameter of 25 mm and a thickness of 2 mm using TA Instruments’ ARES-G2 rotational rheometer (New Castle, UK) according to the ISO 4664:2022 standard [55]. The parameters were: a soak time of 10 s, an angular frequency of 10 rad/s, a logarithmic sweep with strain from 0.005 to 70% s, 20 points per decade, and an initial force of 5 N. The experiment was carried out at room temperature. The storage modulus (G′) describes the elastic properties of the vulcanizate, and it is proportional to the energy retained during its deformation. The loss modulus (G″) presents the viscous properties and is proportional to the amount of work converted into heat during the deformation. The storage and loss moduli values enabled the calculation of the Payne effect according to Formula (11):

(11) $$∆G^{\prime }={G\prime }_{max}-{G\prime }_{min}$$

where G′_max_ is the maximum storage modulus (MPa), and G′_min_ is the minimum storage modulus (MPa).

## 3. Results and Discussion

### 3.1. Characterization of the Morphology and Structure of Biofillers

**Figure 1:**
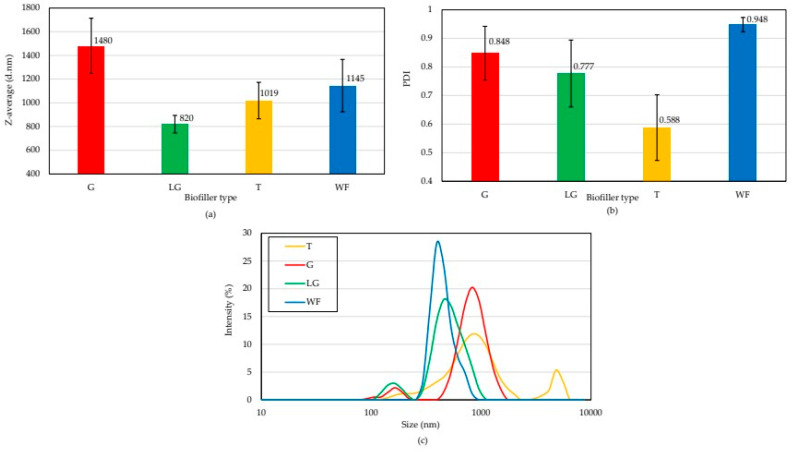
Comparison plot of average hydrodynamic diameter (**a**), particle size dispersion coefficient (**b**), and size distribution of particles (**c**) for ginseng (G), lemon grass (LG), turmeric (T), and wood flour (WD).

The particle size of a filler is one of its most important properties, as it determines the strength of its interactions with the rubber matrix. The particle size of the tested plant-derived fillers was measured using dynamic light scattering (DLS). As shown in Figure 1a, ginseng had the largest average hydrodynamic diameter (1480 nm). Wood flour and turmeric were biofillers with similar average hydrodynamic diameter, reaching 1145 nm and 1019 nm, respectively. Lemongrass had the smallest average hydrodynamic diameter (820 nm). The tested additives also differed in their polydispersity index values (DPI). Figure 1b indicates the most uneven particle size distribution for wood flour (DPI = 0.948). The lowest degree of polydispersity was observed for turmeric (DPI = 0.588), which shows that the turmeric grains were of comparable size. These results are also confirmed in Figure 1c, as the particle size intensity graph shows two peaks for ginseng and lemongrass (~100 nm and 500–1000 nm). Turmeric also had two particle fractions, but in the range of approximately 1000 and 10,000 nm. Interestingly, wood flour only showed a single peak, the most intense of which occurred at approximately 500 nm.

The presence of smaller filler particles increases the active surface area available for interactions at the elastomer-filler interface, which may have a positive effect on the elastomer’s properties. However, other aspects contributing to increased affinity for the elastomeric matrix must also be considered to comprehensively assess the effect of biofillers on biocomposite properties. Therefore, SEM images of natural substances were also obtained.

**Figure 2:**
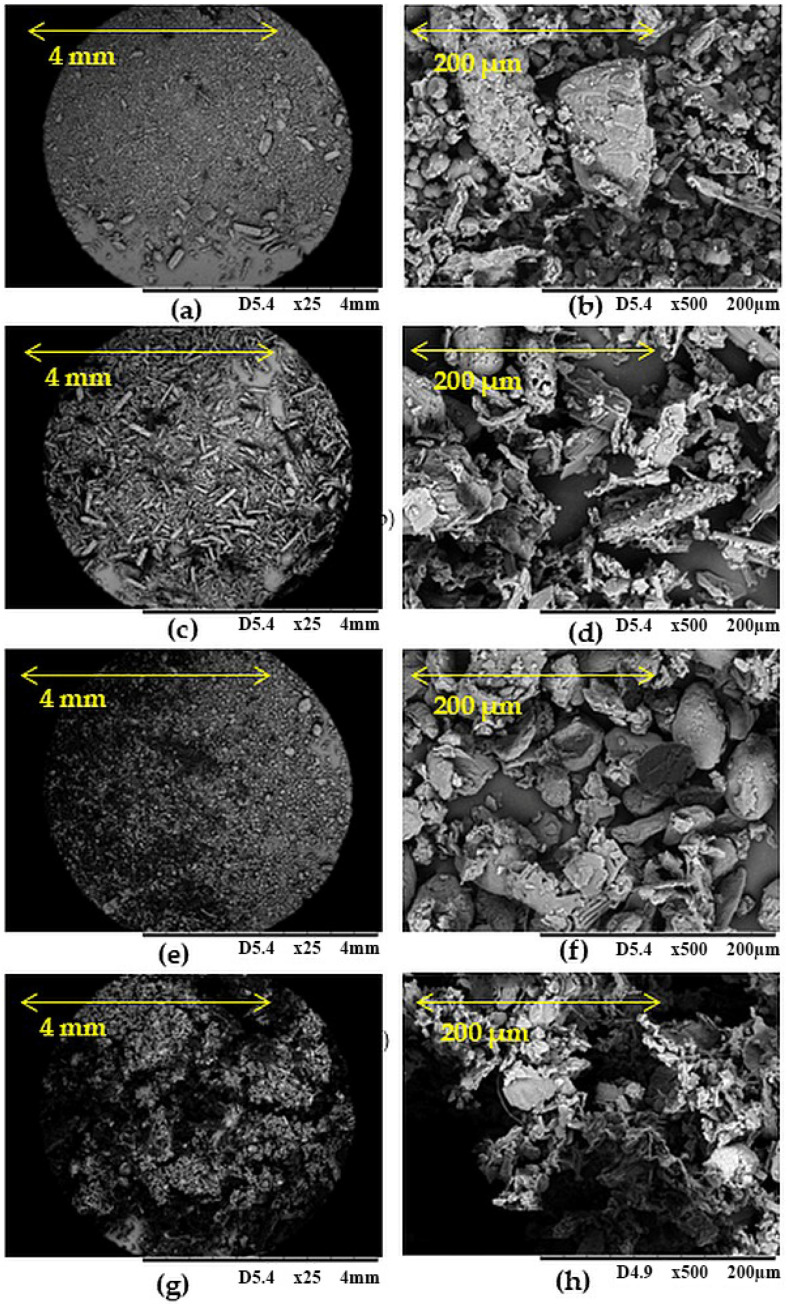
SEM images of ginseng (**a**,**b**), lemon grass (**c**,**d**), turmeric (**e**,**f**), and wood flour (**g**,**h**) at magnification 25× (**a**,**c**,**e**,**g**) or magnification at 500× (**b**,**d**,**f**,**h**).

Figure 2 presents scanning electron microscopy (SEM) images at various magnifications of the tested biofillers. Figure 2a,b show the morphology of ginseng, which indicates that its degree of fragmentation was significant, but large agglomerates of this substance are also visible. Figure 2c,d correspond to lemongrass. They show long, fibrous particles with voids among them. Large, clearly defined turmeric particles are shown in Figure 2e,f. They show uniformly sized particles of this biofiller, confirming previous conclusions based on DLS. A tendency to form larger clusters and agglomerates is observed in SEM images of wood flour (Figure 2g,h). Wood flour grains had an irregular shape, a rougher surface texture, and a more loosely arranged structure. Here, the biofiller grains exhibit the most diverse sizes, as also indicated by analysis of the degree of polydispersity.

**Figure 3:**
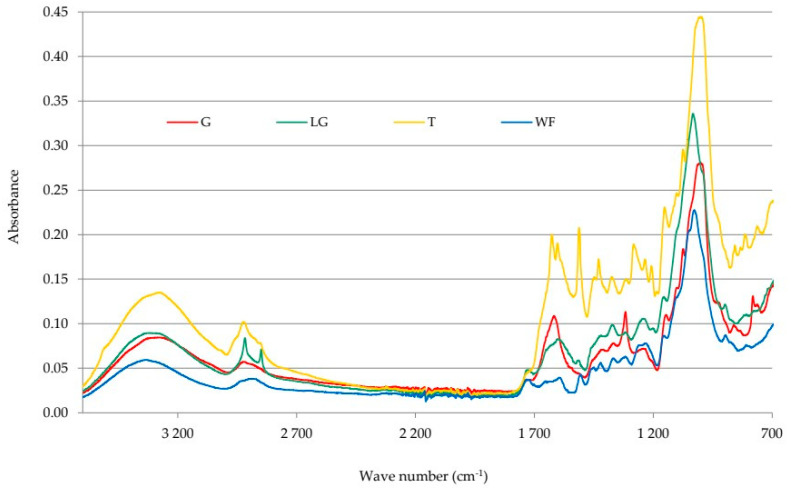
FT-IR spectra of biofillers used in the NR/CR composites: ginseng (G), lemon grass (LG), turmeric (T), and wood flour (WF).

To thoroughly analyze the plant-derived fillers used, infrared spectra were also obtained using Fourier transform infrared spectroscopy (FT-IR). This method detects specific vibrations of molecular bonds in the polymer, enabling the identification of functional groups. Figure 3 shows the spectra of ginseng, lemongrass, turmeric, and wood flour.

The spectra of all biofillers show a broad absorption band in the range of 3270–3380 cm^−1^, indicating stretching vibrations of the hydroxyl group (-O-H). The peak at 2930 cm^−1^ indicates stretching vibrations of the -CH group or deformational vibrations of the -CH_2_ group. The medium-intensity band at 1640 cm^−1^ is most likely associated with water adsorbed in the amorphous region of starches [46]. This peak may also correspond to stretching vibrations of the >C=C< group. In the range of 1450–1200 cm^−1^, bands characterizing deformational vibrations of the O-H bonds of low intensity occur. Stretching and deformational vibrations of the ether bonds (C-O-C) or C-O bonds are confirmed by peaks in the range of 1330–1050 cm^−1^. At a wavenumber of 1000 cm^−1^, a very intense absorption band is visible, indicating stretching vibrations of the -O-H group. This band is coupled to the stretching vibrations of adjacent C-C bonds and may be present in phenolic groups. Furthermore, the peak at 780 cm^−1^ is responsible for out-of-plane O-H deformation vibrations [56]. All of the described peaks are present in the spectrum of each biofiller tested, confirming their very similar (but complicated) chemical structure, resulting from the chemical composition of these substances. Each of them contains, among other things, fragments typical of cellulose or aromatic lignin groups. Some differences can only be observed in the spectrum of turmeric, compared to the spectra of the other fillers. The spectrum of turmeric containing polyphenols shows numerous low-intensity bands in the range of 1500–1300 cm^−1^, which may indicate in-plane deformation vibrations of the methylene and methyl groups and confirm that this region corresponds to complex stretching and deformation vibrations of the molecular skeleton. Interpretation of these bands is significantly hampered by numerous overlapping bands that cannot be associated with specific, distinct structural elements of the chemical compound.

### 3.2. Evaluation of the Morphology of NR/CR Vulcanizates Before and After Filling with Bio-Fillers

The purpose of using scanning electron microscopy (SEM) was to analyze the structure of NR/CR vulcanizates and the impact of biofillers and their modification with silane on their dispersion in the tested blends. The physical and chemical properties of cross-linked elastomers or their blends significantly depend on the filler particles’ degree of dispersion and aggregation. In addition, the SEM analysis provided information on the miscibility of the components in NR/CR composites. Therefore, understanding the morphology of the elastomer material was very important.

**Figure 4:**
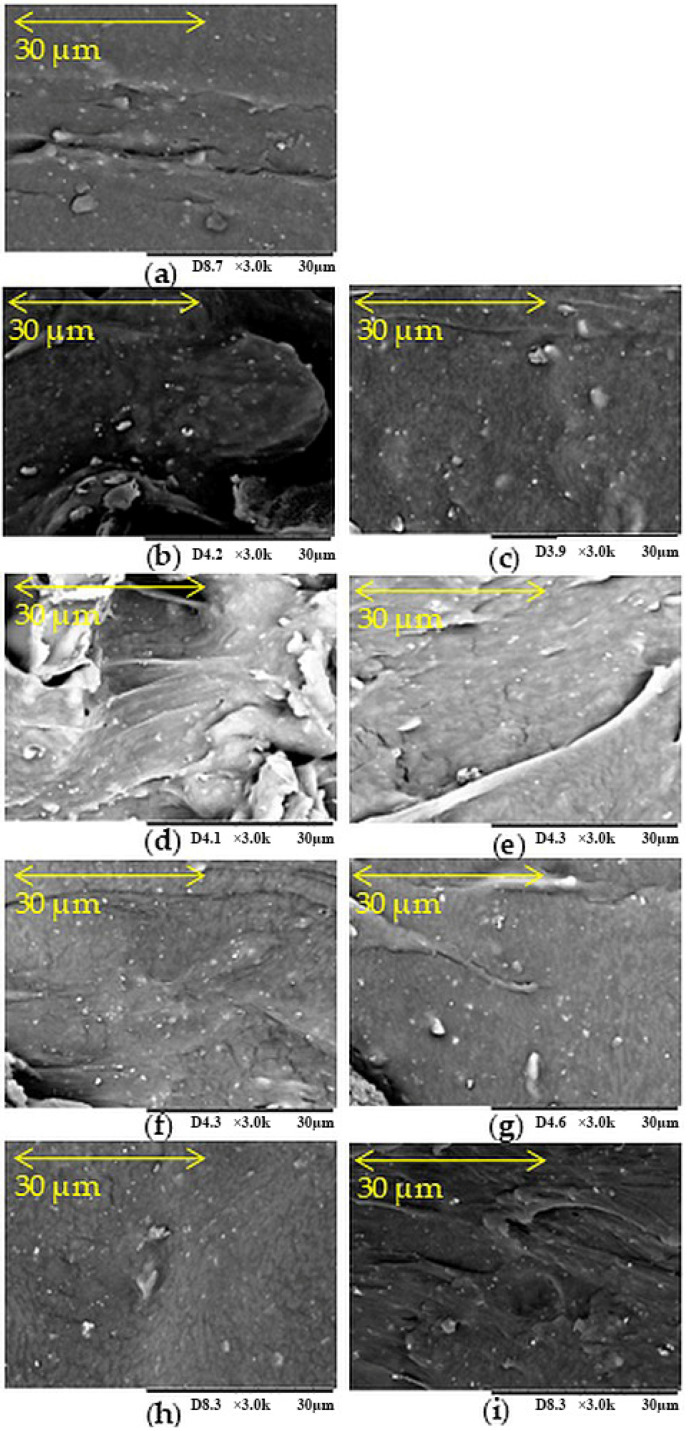
SEM photographs of the cross-section of samples: (**a**) unfilled NR/CR, (**b**) NR/CR filled with ginseng (G), (**c**) NR/CR filled with modified ginseng (G-OTS), (**d**) NR/CR filled with lemongrass (LG), (**e**) NR/CR filled with modified lemongrass (LG-OTS), (**f**) NR/CR filled with turmeric (T), (**g**) NR/CR filled with modified turmeric (T-OTS), (**h**) NR/CR filled with wood flour (WF), (**i**) NR/CR filled with modified wood flour (WF-OTS); magnification: 3000×.

The cross-section morphologies of the tested NR/CR vulcanizates at a 3k magnification are shown in Figure 4. The SEM image of unfilled NR/CR vulcanizate (Figure 4a) confirms their limited miscibility. It shows a rougher surface with a corresponding tortuous path, indicating that chloroprene and natural rubber did not form a homogeneous system after cross-linking. Incorporating biofillers into such a composition led to different morphologies of the tested materials, depending on the plant substance used. In Figure 4b, showing a photo of the NR/CR vulcanizates filled with ginseng, large inhomogeneous areas, including empty fragments, are visible. An even more inhomogeneous structure can be seen in Figure 4d, which concerns the vulcanizate filled with lemongrass. Even fibrous structures are visible here, probably resulting from the structure of this plant. Lemongrass tends to form agglomerates and aggregates, as Figure 4d suggests.

A more uniform morphology for the vulcanizate containing turmeric was observed (Figure 4f). In this case, only small folds were visible, and the bio-filler used was dispersed quite evenly. However, the most uniform morphology was confirmed for the NR/CR/wood flour composition (Figure 4h), for which only small aggregates of the low-molecular-weight substance were visible. The composition was characterized by the best dispersion of low-molecular-weight components in the elastomeric matrix, and the system observed in the SEM image was much more uniform than the unfilled vulcanizate. All filled vulcanizates were chosen to investigate the effect of n-octadecyltrimethoxysilane on the filler and morphology of the sample. Figure 4c,e,g,i represent surfaces of composites with bio-fillers modified with n-octadecyltrimethoxysilane. Analyzing the presented SEM photos, it was visible that the incorporation of silane into the NR/CR/biofiller composites resulted in better dispersion and smaller filler particles in the elastomeric matrix. A particularly significant improvement in the proper dispersion of ingredients in vulcanizates can be observed when using silane-modified ginseng, lemongrass, and curcumin. Proper dispersion of used biofillers in the elastomeric matrix was essential for the correct effectiveness of the reinforcing action of the filler. This was achieved by adding silane to the NR/CR compositions.

### 3.3. Cross-Linking Results of Unfilled and Filled NR/CR Blends

Cross-linking (as vulcanization) is the chemical, permanent connection of elastomer macromolecules by the cross-links, leading to the network. The type and amount of curing agents used determine the functional properties of the vulcanizates [57]. The presence of specific functional groups with unique chemical activity is necessary for the cross-linking of the elastomers. The chemical structure of the elastomer determines the ability of the respective cross-linking agent to react with the rubber at a controlled rate. Many other factors, including the type of cross-linking substance used, influence the vulcanization process, such as the type of filler used. Thus, the rheological properties of NR/CR blends were studied to determine the effect of biofillers and to describe the vulcanization process. The rheometric parameters are presented in Table 2. When vulcanization sets in, the most obvious changes in a rubber mix are an increase in stiffness and an increase in the elastic component of its viscoelastic deformation. The cross-linking parameters indicate the viscosity of the blends and determine the scorch time (t_02_), vulcanization time (t_90_), minimum torque (T_min_), maximum torque (T_max_), and maximum torque increment (ΔT_max_). A thorough examination of the cross-linking kinetics allows us to select the parameters of the vulcanization process in such a way that the final products have the best possible properties.

The analysis of the study’s results on the cross-linking kinetics of elastomers shows that the plant-derived natural fillers used significantly influence the cross-linking process. One of the vulcanization parameters that depends on the type of elastomers used in the blend is the scorch time, which defines the safety of elastomer processing at higher temperatures. The longer the scorch time is, the greater the processing safety of the rubber compound is. All the filled NR/CR blends achieved shorter scorch time than the blend without any filler because the t_02_ value for unfilled composites was equal to 1.63 min, and using natural filler increased this parameter by about 6–20%. The incorporation of n-octadecyltrimethoxysilane as a modifier of the fillers used led to a further reduction in the scorch time. In the case of the composition containing LG-OTS, the scorch time was only 1.14 min, but it was still such a t_02_ value that guarantees the safe processing of such a rubber mix and ensures the appropriate plasticity necessary for the correct course of the entire processing.

From a technological point of view, an important parameter is the vulcanization time, defined by the t_90_ parameter, which corresponds to 90% exhaustion of the curing agent necessary to generate cross-links between macromolecules. The t_90_ value is assumed to be the time required for a 90% increase in torque and is perceived as the proper cross-linking time of the rubber compound. In this work, the shortest cure time (or vulcanization time) was characteristic for the NR/CR blend filled with turmeric (t_90_ = 3.69 min), and the longest cure time (t_90_ = 5.27 min) was achieved for the NR/CR blend containing wood flour. The modification of fillers with n-octadecyltrimethoxysilane resulted in an extension of the vulcanization time in each tested blend. Cure time is an important economic aspect; therefore, such a short cure time achieved for the NR/CR blends is their great advantage.

Another measured parameter was the minimum torque, which was responsible for the viscosity of NR/CR blends. The tested blends had a similar value of this parameter, so it can be stated that none of the tested fillers significantly affected the viscosity. The minimum torque value of the unfilled blend was 0.23 dNm, while the compositions containing unmodified biofillers had the T_min_ parameter of 0.24–0.25 dNm. It is worth emphasizing that the incorporation of n-octadecyltrimethoxysilane as a filler modifier to the NR/CR blends slightly increased the viscosity of these compounds, which was confirmed by higher values of the minimum torque (T_min_ = 0.27–0.29 dNm).

**Figure 5:**
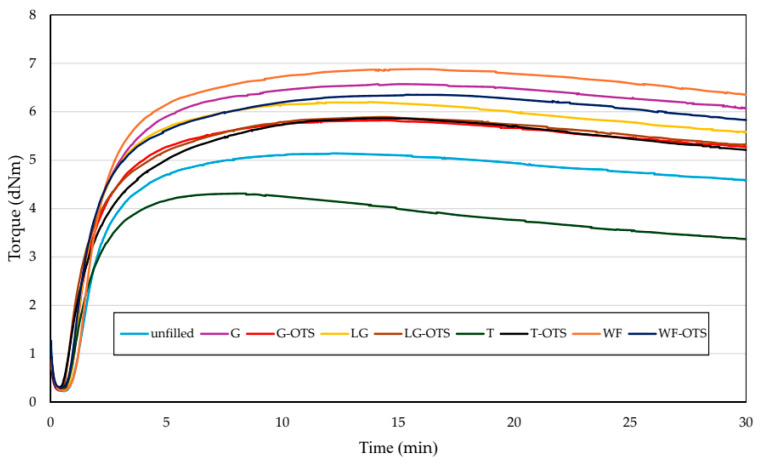
Vulcanization curves of unfilled and filled NR/CR (50/50 by wt.) blends.

Interpreting rheometric parameters, particularly the maximum torque increment (∆T_max_), is an important tool for assessing the degree of cross-linking in elastomeric materials. A high ∆T_max_ value indicates a greater difference between the initial and maximum torque, which directly correlates with the density of the cross-link network formed during the vulcanization process. The highest ∆T_max_ values in NR/CR blends with ginseng (∆T_max_ = 6.63 dNm) or wood flour (∆T_max_ = 6.33 dNm) may indicate the active participation of these natural fillers in the formation of the elastomeric network structure, likely through physicochemical interactions between their active ingredients and the elastomeric matrix. In turn, the reduction in ∆T_max_ after using silane (except in the case of turmeric) may suggest that this modification limits the formation of the sulfide bridges or affects the dynamics of the cross-linking reaction—potentially by altering the dispersion or interfacial interactions. The case of turmeric is interesting and requires further analysis—perhaps the bioactive components present in turmeric (e.g., curcuminoids) enter into synergistic reactions with the silane or rubber matrix, supporting the cross-linking process. All samples had rheometric curves with a reversion (Figure 5). This is an undesirable and competitive phenomenon in the rubber vulcanization process, as it decomposes the crosslinks formed during the actual cross-linking stage, leading to a reduction in the degree of cross-linking and a deterioration in the final properties of the vulcanizates. It is worth noting that the highest reversion was observed for the unfilled NR/CR blend. The incorporation of biofillers significantly reduced the susceptibility of the tested compositions to reversion. Based on the results obtained, the vulcanization time for all blends was set at 5 min.

### 3.4. Influence of Biofillers on Thermal Analysis of NR/CR Composites

**Figure 6:**
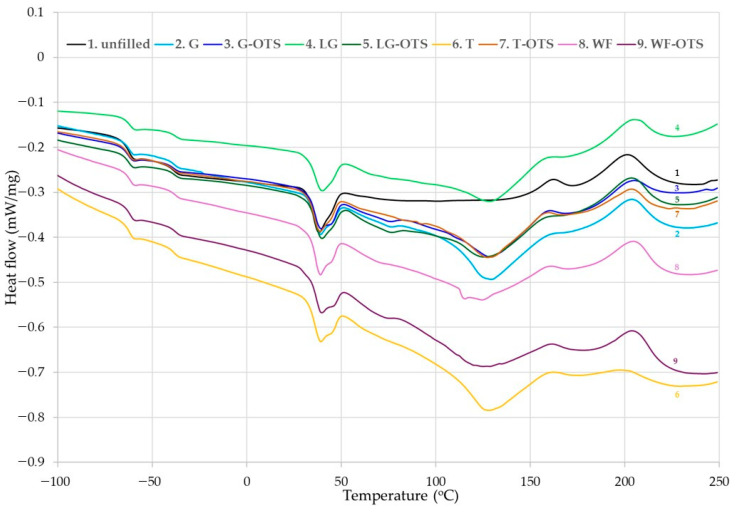
Differential scanning calorimetry (DSC) curves of unfilled and filled NR/CR (50/50 by wt.) blends.

The differential scanning analysis can be a confirmation of interelastomer reactions between the non-polar natural rubber and polar chloroprene rubbers. Elastomeric blends most often form immiscible systems. The miscibility of several elastomers is designated by the polarity of the rubbers and their glass-transition temperatures. Interelastomer reactions occurring between NR and CR can lead to improved homogeneity and miscibility of the tested vulcanizates. The miscibility of two dissimilar elastomers can also be improved by suitable fillers or their modification. It was also expected that the presence of n-octadecyltrimethoxysilane in the tested compositions would be of great importance in this aspect. In the DSC curves (Figure 6), it can be observed that there are two glass-transition temperatures, and each of them corresponds to the individual phases of the blend, i.e., natural or chloroprene rubber. The glass-transition temperature for natural rubber was in the range from −62.8 °C to −61.8 °C, and for chloroprene rubber, T_g_ was −38.7 °C to −37.7 °C. However, the observed slight changes in the glass transition temperatures of both elastomers may indicate the presence of interactions and increased compatibility between natural and chloroprene rubbers.

The DSC curves also show the changes occurring in the NR/CR blends in a given temperature range. The enthalpy determined for the vulcanization range based on the changes occurring in the sample during heating is related to the degree of cross-linking. The higher the enthalpy value, the greater the degree of cross-linking of the filled elastomeric compositions. For comparison, Figure 6 and Table 3 also show the results for the sample without biofiller.

The DSC analysis results provide an important complement to the rheometric data, enabling direct observation of thermal effects accompanying chemical reactions, such as cross-linking of elastomeric blends. The presence of an exothermic peak in the temperature range of 157–219 °C for the NR/CR/G composition indicated an intense cross-linking reaction. The high enthalpy of this transformation (∆H = 32.92 J/g) indicated significant energy involvement in the cross-link formation, which correlated with the previous observation of the highest ∆T_max_ in the rheometric analysis of the same composition. Therefore, the DSC data confirmed that the NR/CR/G composition was characterized by the highest degree of cross-linking among the tested samples. A high enthalpy value (26.71 J/g) was also observed for the NR/CR/LG-OTS sample, where the transition temperature range was slightly shifted (166–221 °C). In this case, the modification of the lignocellulosic filler with silane (OTS) probably improved its compatibility with the elastomeric phase. The use of binding silanes (including long-hydrocarbon OTS) enabled the formation of chemical bridges between the hydroxyl groups present on the lemongrass surface and the rubber matrix, increasing the efficiency of the cross-linking process. Furthermore, improved filler dispersion in the elastomeric blend probably increased the availability of active cross-linking sites. In turn, the lowest enthalpy of transformation (16.93 J/g) observed for the unfilled NR/CR composite was consistent with the lower degree of cross-linking in this blend. The absence of functional fillers limited the number of active places that could initiate or accelerate the cross-linking reaction. This result was confirmed by the rheometric data, where this sample had the lowest ∆T_max_. It is worth noting that both DSC analysis and vulcametric parameters consistently indicated the influence of the type and nature of the biofiller on the efficiency of the vulcanization process, the intensity of the cross-linking reaction, and the final properties of the elastomeric composites.

The DSC thermogram presents all reactions occurring in an indicated temperature range. It is obvious that during the heating of the NR/CR blends, many reactions occur; therefore, it is very difficult to measure the enthalpy for a specific reaction. On the DSC curves of all NR/CR composites, a visible endothermic peak at 45 °C corresponded to the crystalline phase of chloroprene rubber. This phenomenon is a typical behavior of this elastomer, because it can crystallize during stretching and become an amorphous compound during heating.

### 3.5. Swelling Behavior of Unfilled and Filled NR/CR Vulcanizates

The swelling process of rubber products in the presence of solvents is one of the crucial parameters assessing vulcanizates’ chemical resistance and quality. This is undesirable, as it leads to reduced mechanical strength and can result in degradation of the material’s functional properties. The main factor influencing the intensity of swelling is the degree of cross-linking—the greater the degree of cross-linking, the lower the material’s ability to absorb solvents, and therefore the better its structural stability. The equilibrium swelling test was carried out in two organic solvents: toluene and hexane. Table 4 presents the results of the tests of the prepared samples.

In toluene, the NR/CR vulcanizate filled with ginseng demonstrated the highest chemical resistance. The highest degree of cross-linking (α_c_ = 0.257) indicated an effective vulcanization process, which translated into the lowest equilibrium swelling (Q_v_^T^ = 3.89 mL/mL), compared to the other samples. This means that this composite resisted the effects of toluene most effectively, making it potentially more durable in environments containing aromatic solvents. In contrast, the worst results were recorded for the NR/CR/turmeric sample, where the cross-linking degree was the lowest (α_c_ = 0.173) and the volumetric swelling degree was the highest (Q_v_^T^ = 5.78 mL/mL). This indicates weaker spatial bonding in the elastomeric network, allowing toluene to penetrate the rubber structure more easily and cause swelling.

Changing the solvent to hexane revealed a slightly different picture of the samples’ chemical resistance. The composite with turmeric also exhibited the highest swelling degree in hexane (Q_v_^T^ = 1.62 mL/mL). This suggests that regardless of the solvent (aromatic or aliphatic), the low degree of cross-linking and the specific interaction of turmeric with the elastomer negatively impacted the material’s stability. The best resistance to hexane was demonstrated by the NR/CR vulcanizates containing silane-modified wood flour, for which the Q_v_^H^ was only 1.14 mL/mL. This indicates that the use of the modified biofiller effectively limited solvent penetration into the rubber structure, likely by improving adhesion between the organic elastomer phase and the inorganic filler and by enhancing the cross-linking effect.

**Figure 7:**
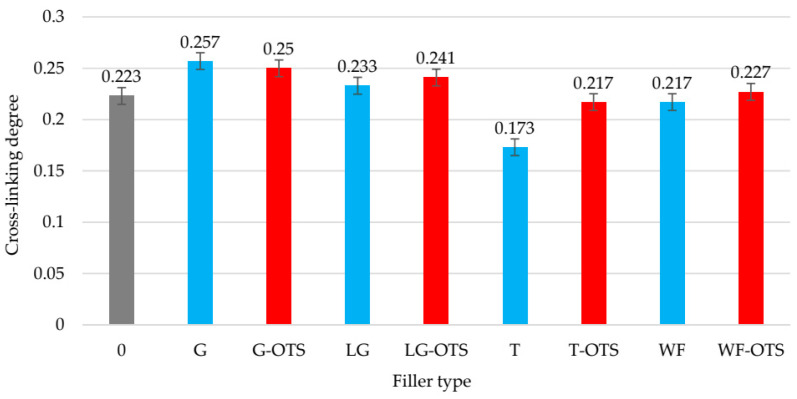
The degree of cross-linking of unfilled and filled NR/CR (50/50 by wt.) vulcanizates.

Analysis of all samples showed that the addition of silane in most cases reduced the swelling of the vulcanizates, which can be explained by its function as a coupling agent, improving the compatibility between the elastomer matrix and the filler. Silanes can also participate in cross-linking reactions, increasing the degree of cross-linking. In Figure 7, a summary of vulcanizates’ degrees of cross-linking (α_c_) is presented.

### 3.6. Tensile Properties of Unfilled and Filled NR/CR Vulcanizates Before and After Thermo-Oxidative Aging

The cross-linked elastomeric composites are characterized by unique mechanical properties, such as a high elasticity, the ability to undergo reversible deformation, and good resistance to tension and compression. Thanks to the cross-linked polymer structure, rubber can be repeatedly deformed and return to its original shape without permanent damage. The most important mechanical parameters of rubber include tensile strength, elongation at break, modulus of elasticity, hardness, tear resistance, and hysteresis. These properties can be modified by selecting the type of rubbers, additives (e.g., fillers), and the degree of cross-linking. The mechanical properties of the vulcanizates are important in technical applications requiring a durability, flexibility, and resistance to external forces. The mechanical properties of the NR/CR vulcanizates were determined by means Zwick machine (Table 5). From the obtained results, it was found that the type of biofiller used and its modification affect the tensile properties of the compounds tested. The first considered parameter is the stress at a relative elongation of 100%, 200%, or 300% (S_e100_, S_e200_, and S_e300_, respectively). The stress at elongation of 100% is critical because it determines the stiffness of the tested samples. Samples with natural fillers had higher stresses at 100% elongation (from 1.23 to 1.40 MPa) compared to unfilled vulcanizate (S_e100_ = 0.79 MPa). The exception was the vulcanizate filled with turmeric, because in this case lower S_e100_ value (0.71 MPa) was observed. It indicated the lowest stiffness of such an elastomeric product. The addition of silane increased the values of the stress at 100% elongation of the vulcanizates, except for the samples with ginseng.

The most important mechanical parameter assessed in this study is the tensile strength (TS_b_), defined as the maximum stress a material can withstand before breaking during stretching. This parameter is essential in assessing the durability and overall performance of the vulcanized rubber composites, particularly in applications where mechanical loading is expected. Among the tested samples, the vulcanizate filled with ginseng exhibited the highest tensile strength, reaching a value of TS_b_ = 7.22 MPa. This may indicate favorable interactions between the particles of the ginseng and the elastomer matrix, which translates into improved stress transfer and overall structural cohesion of the material. It is worth emphasizing, however, that most of the tested samples exhibited relatively similar levels of tensile strength—the TS_b_ values for these materials fell within a relatively narrow range of 6.3 to 7.2 MPa. This suggests that the use of various plant additives did not drastically weaken the mechanical structure of the composite, and some may even exhibit a strengthening effect. The exception was the sample containing turmeric, whose tensile strength was significantly lower, reaching only TS_b_ = 3.94 MPa. Such a significant decrease in this parameter may indicate unfavorable interactions between the turmeric components and the elastomer matrix or disturbances in the vulcanization process, resulting in decreased mechanical properties. The presence of such a filler led to a weakening of the vulcanizate’s bond network, which directly translated into a reduction in the material’s tensile strength. In summary, the tensile strength measurement results indicated that using biofillers, such as ginseng, can improve the mechanical properties of vulcanizates. However, caution should be exercised when selecting biofillers, as not all of them positively impact the structure and durability of the material. In most cases, modification of the plant fillers with silane led to the production of vulcanizates with slightly higher mechanical strength (except for the sample filled with ginseng). In the case of the NR/CR/turmeric composite, the tensile strength improved by over 40% (from 3.91 MPa to 5.53 MPa). At the same time, the obtained results confirmed that biofillers did not significantly affect the elongation at break of the tested vulcanizates.

The behavior of the vulcanizates over time, particularly their durability and stability of mechanical properties, is crucial for their practical application. Premature and undesirable aging of elastomeric materials, resulting from the action of external factors such as temperature, oxygen, UV radiation, and moisture, is one of the most common defects limiting the service life of rubber products. Aging processes lead to degradation of the chemical structure of the elastomer network, which in turn leads to a deterioration of physical and mechanical properties such as elasticity, strength, and deformation resistance. Therefore, it is crucial to develop and implement technological solutions that allow for the production of vulcanizates characterized by increased resistance to factors that accelerate aging, which translates into a longer service life of finished products, particularly in applications exposed to unfavorable environmental conditions. This study assessed the resistance of NR/CR vulcanizates filled with plant fillers to the thermo-oxidative aging process. For this purpose, samples were exposed to elevated temperatures (70 °C) under oxidizing conditions for seven days. This treatment aimed to simulate long-term use of the material under accelerated aging conditions typical of industrial or operational environments. The impact of the aging process was assessed by comparing mechanical properties before and after exposure to aging factors. The following parameters were analyzed: stress at 100% elongation, tensile strength, and elongation at break, which are important indicators of the quality and structural integrity of elastomers. The results are presented in Table 5.

Based on the obtained data, it can be seen that most of the tested samples exhibited relatively good resistance to thermo-oxidative aging, although differences in the level of retention of mechanical properties were clearly dependent on the type of filler used. The unfilled NR/CR vulcanizate showed a slight increase in all stress parameters (Se_100_–Se_300_) after aging, with a simultaneous increase in tensile strength by approximately 6% (from 6.31 MPa to 6.70 MPa) and a decrease in elongation at break by approximately 12% (from 994% to 870%). This suggests partial reorganization of the elastomer network, leading to material stiffening. The vulcanizate with the addition of turmeric demonstrated the greatest resistance to aging, with the TS_b_ after aging increasing from 3.94 MPa to 4.56 MPa (an increase of approximately 16%). However, it should be noted that this parameter remained the lowest among all samples. But its increase after exposure to elevated temperatures, which we encountered during the aging process, was most likely related to the properties of turmeric. This compound exhibits strong antioxidant properties resulting from the presence of phenolic groups, which effectively capture hydroxyl radicals generated during the oxidation of natural rubber or chloroprene chains. Additionally, turmeric components are capable of chelating transition metal ions, limiting their contribution to hydroxyl radical generation. The system of conjugated double bonds present in turmeric ensures effective absorption of UV radiation, which reduces the intensity of photooxidation. Ozone cracking is also likely partially inhibited, resulting from turmeric’s reactivity to ozone and oxygen radicals. The integrated antioxidant, photoprotective, and polymer structure-stabilizing effects lead to a significant increase in the aging resistance of NR/CR compositions, indicating turmeric’s potential as an eco-friendly, functional biofiller in elastomer composites.

**Figure 8:**
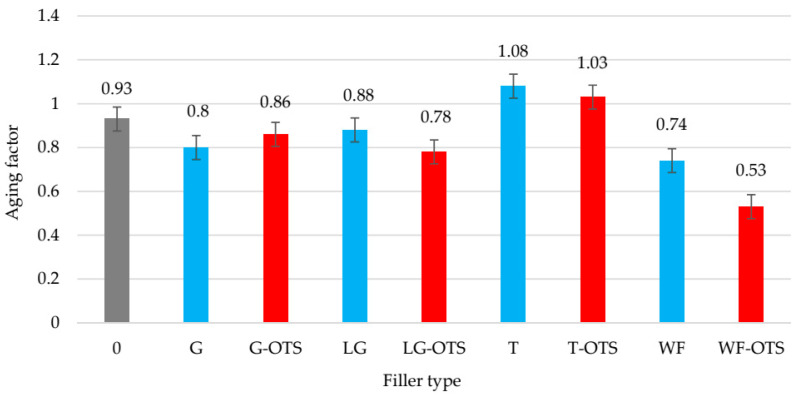
Aging factors of unfilled and filled NR/CR (50/50 by wt.) vulcanizates.

The sample with the addition of wood flour demonstrated the lowest resistance to thermo-oxidation, with the TS_b_ dropping from 6.29 MPa to 5.53 MPa (a 12% decrease) after aging. The aging factor in this case was 0.74. Adding silane-modified wood flour to the NR/CR composition resulted in even lower resistance to thermo-oxidative aging (AF = 0.53). In this case, strength decreased by approximately 25%, and elongation decreased by approximately 30%. Overall, the use of silane reduced the aging resistance of the vulcanizates. The only exception was the sample filled with modified ginseng. In summary, the research results clearly indicate that the type of plant-based filler significantly influences the resistance of NR/CR vulcanizates to thermo-oxidative aging. Therefore, selecting the appropriate additive can effectively extend the elastomer product’s service life under demanding environmental conditions. Figure 8 shows that the aging factors calculated from Formula (9) were high for all the produced NR/CR/biofiller vulcanizates.

### 3.7. Hardness of Unfilled and Filled NR/CR Vulcanizates

**Figure 9:**
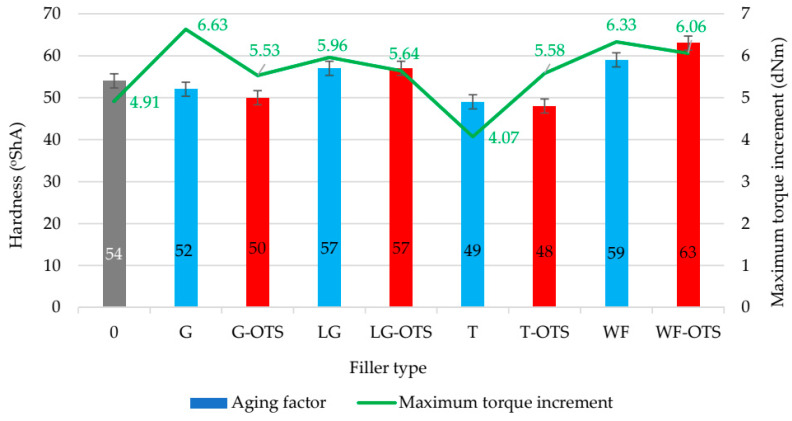
Hardness vs. maximum torque increment of unfilled and filled NR/CR (50/50 by wt.) vulcanizates.

In the case of rubber materials, hardness is directly related to the degree of cross-linking, e.g., the higher the degree of cross-linking, the greater the material’s hardness. Figure 9 presents the results of Shore A hardness measurements for CR/NR vulcanizates containing the tested biofillers before and after silane modification. The hardness values for the analyzed samples fell within a relatively narrow range from 48 °ShA to 63 °ShA, indicating a generally similar level of cross-linking for the NR/CR compositions, although with clearly noticeable differences depending on the filler type and modification. The sample containing modified wood flour (NR/CR/WF-OTS) exhibited the highest hardness (63 °ShA), suggesting that both the filler itself and its silane modification significantly increased the material’s cross-linking. This is confirmed by previous rheometric measurements, in which this sample exhibited the greatest increase in maximum torque (∆T_max_), indicating strong interactions within the elastomeric matrix.

In contrast, the vulcanizates filled with turmeric (NR/CR/T and NR/CR/T-OTS) exhibited the lowest hardness (48 °ShA and 49 °ShA), confirming the lowest degree of cross-linking among the filled materials tested (∆T_max_ = 4.07 dNm, α = 0.173; for NR/CR/T). The low HA value of this sample was consistent with its high elasticity and low tensile stress. These results indicated weaker interactions between the elastomer matrix and the turmeric particles, even after modification with OTS. In most cases, modification of the biofiller with silane resulted in a decrease in the hardness of the vulcanizates. This may indicate that the silane limits particle agglomeration or reduces their stiffness, which reduces the overall hardness of the system. An exception to this rule was the previously mentioned sample with modified wood flour, in which the modification had the opposite effect—it led to an increase in hardness, most likely due to a stronger bond between the filler phase and the rubber matrix.

### 3.8. Tear Resistance of Unfilled and Filled NR/CR Vulcanizates

The tear resistance (T_s_) is one of the essential mechanical properties of rubber composites, particularly important in applications where the material is exposed to tensile forces, which cause crack initiation and propagation. This property determines the operational durability of rubber products and their resistance to mechanical damage. Table 6 summarizes the tear strength measurement results for the tested vulcanizates containing various biofillers, both unmodified and silane-modified.

The data analysis showed that the vulcanizates containing unmodified and modified wood flour exhibit the highest tear resistance, with maximum tear strength values (T_s/max_) reaching 9 and 13 N/mm, respectively. This result was significantly higher than for the other samples. It can be assumed that wood flour, thanks to its fibrous structure and ability to form hydrogen and covalent bonds (after modification), effectively increased the material’s resistance to crack propagation. At the other end of the spectrum, the sample containing turmeric achieved the lowest tear strength values: T_s/med_ = 3.51 N/mm, T_s/max_ = 4.18 N/mm. This means that the tear resistance of this sample was lower compared to the unfilled sample. This result may be due to the weak adhesion of the turmeric particles to the rubber matrix, as well as the lack of a structure supporting the transfer of mechanical loads. Turmeric, as a fine-grained organic substance, may act more as a plasticizing agent than a reinforcing agent, as previously indicated (see Table 5).

Silane modification of the fillers showed a varied effect on tear resistance. In the case of the samples containing ginseng or lemongrass, a decrease in tear strength was observed after modification with OTS (a decrease of 7% and 23%, respectively). This may indicate that silane modification was not effective in these cases—the filler surface likely did not fully compatibilize with the rubber matrix, and the presence of silane may even have disrupted existing interfacial interactions. In the case of the samples containing turmeric and wood flour, the modification resulted in a significant improvement in mechanical properties. The NR/CR/T-OTS vulcanizate achieved a maximum tearing force of 6.16 N/mm, a significant increase compared to its unmodified counterpart (4.18 N/mm). This indicated that adding silane improved the compatibility of the turmeric with the rubber matrix, likely by incorporating additional chemical bonds. The most dramatic increase in tear resistance occurred in the NR/CR vulcanizate filled with WF-OTS, further emphasizing the effectiveness of this type of filler and the benefits of its chemical modification.

### 3.9. Hysteresis Losses and Mullins Effect of Unfilled and Filled NR/CR Vulcanizates

**Figure 10:**
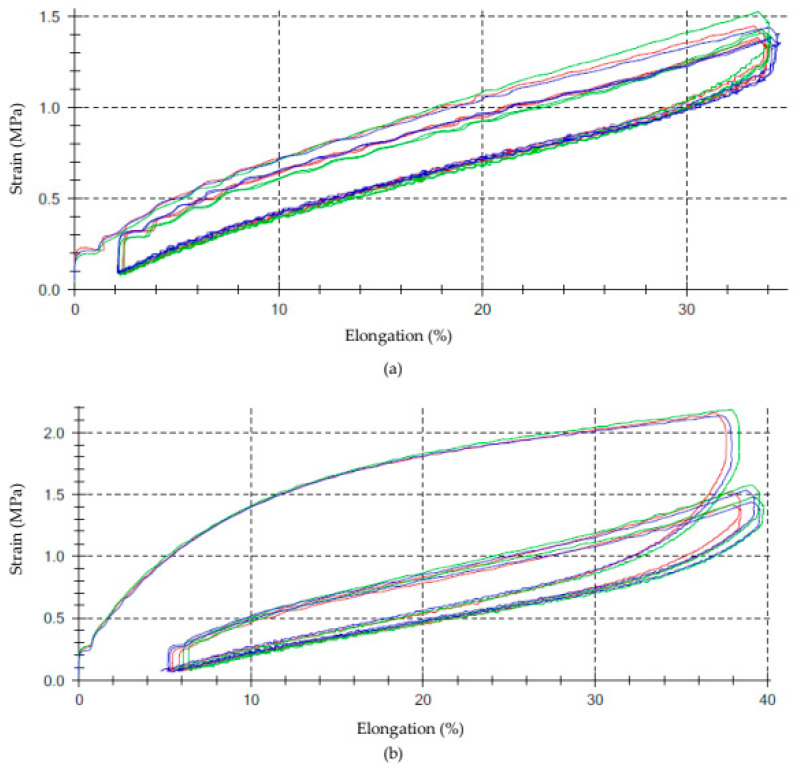
Hysteresis loops of NR/CR (50/50 by wt.) vulcanizate: (**a**) unfilled, (**b**) filled with wood flour.

Hysteresis in elastomeric materials is a phenomenon whereby, during cyclic deformation of the material (stretching and relaxation), not all of the supplied mechanical energy is recovered. Some of this energy is dissipated as a heat due to internal molecular friction, the breaking of weak interfacial bonds, and structural changes in the material. As a result, the strain-elongation graph (the so-called mechanical cycle curve) forms a loop called a hysteresis loop. The area of this loop corresponds to the energy losses that are not recovered during deformation. Figure 10 compares the hysteresis loops of an unfilled NR/CR vulcanizate and a vulcanizate filled with wood flour. Table 7 presents the hysteresis results for all tested NR/CR compositions.

In elastomeric materials containing fillers, the hysteresis is particularly pronounced and is associated with the so-called Mullins effect. The Mullins effect (E_M_) is associated with a reduction in stress during a series of identical deformations of vulcanizates, resulting from the destruction of bonds between the elastomer and the filler and the interactions between filler molecules. The higher the E_M_ value, the greater the difference in the work required to stretch the sample in the first and subsequent stretching cycles, resulting in greater stress relaxation. The Mullins effect also depends on the degree of filler dispersion. When the filler is poorly dispersed, large agglomerates occur, the structures of which are destroyed by the series of deformations, leading to the stress relaxation and greater Mullins effect.

The unfilled NR/CR vulcanize exhibited the lowest hysteresis losses (∆W_i_ = 34.1 Nmm) and the lowest Mullins effect (E_M_ = 10.5%), which was represented by the smallest hysteresis loop visible in Figure 10a. Among the filled vulcanizates, the lowest hysteresis loss and the lowest Mullins effect were recorded for the composition filled with turmeric (∆W_i_ = 77.2 Nmm, E_M_ = 38.2%). In this case, the previously described strength and tear properties indicated weak interactions between the elastomeric matrix and turmeric; hence, the hysteresis results support this conclusion. The highest mechanical energy losses and the highest Mullins effect were observed for the sample NR/CR filled with wood flour, both unmodified (∆Wi = 164.7 Nmm, E_M_ = 52.8%) and silane-modified (∆Wi = 191.5 Nmm, E_M_ = 57.0%). This was also confirmed in Figure 10b, which clearly shows very large hysteresis loops. These results indicated significant strength of interactions between the wood flour and the elastomer, resulting in greater work required to stretch the sample and greater energy losses. Vulcanizates filled with wood flour (especially modified wood flour), therefore, required significant energy input during stretching, and much of this energy was converted into thermal energy due to intense stress relaxation. It is also worth noting that adding silane into the biofillers used in the tested materials enhanced the Mullins effect, thus confirming the positive role of OTS and its impact on stronger interactions between the plant-derived fillers used and the elastomeric matrix.

### 3.10. Dynamic Properties and Payne Effect of Unfilled and Filled NR/CR Vulcanizates

The Payne effect is an increase in the storage (G′) and loss (G″) modulus caused by the presence of a filler in the vulcanizate and its ability to form networks. This phenomenon depends on various factors, including the type and concentration of the filler, its dispersion, and the type of rubber. Filler-elastomer interactions relate to the compatibility of these systems and the formation of “bound rubber,” which refers to trapped rubber fragments within the filler agglomerates. Occlusion of rubber increases the filler’s effective volume, leading to a decrease in the elastic modulus and a more pronounced Payne effect. The strongest influence on the filler’s reinforcing ability comes from interactions that cause filler particles to attract each other. Larger particles produce a stronger Payne effect. The storage modulus (G′) indicates the material’s capacity to store energy, while the loss modulus (G″) indicates its ability to dissipate energy. A higher loss modulus means more mechanical energy is converted into heat, enhancing damping properties. The G″ value increases with the rate of structure destruction and reformation, which is related to internal friction in the filled vulcanizates. In Table 8, the results of the tests on the prepared samples are presented.

The evaluation of the dynamic properties of NR/CR vulcanizates can highlight many features of a given material. The higher the degree of cross-linking, the higher the observed value of the storage modulus, and the lower the loss modulus. As expected, the lowest storage modulus (G′_max_ = 0.165 MPa) and Payne effect (∆G′ = 0.151 MPa) were observed for unfilled NR/CR vulcanizate. A relatively small Payne effect characterized vulcanizates with well-dispersed filler. A high Payne effect for the NR/CR vulcanizate filled with a turmeric (∆G′ = 0.732 MPa) or wood flour (∆G′ = 0.677 MPa) suggested poor dispersion of these fillers and a large number of agglomerates or strong rubber-filler interactions. The lowest values of storage modulus and loss modulus were observed for vulcanizates filled with ginseng. For this composite, the Payne effect was the smallest (∆G′ = 0.300 MPa for NR/CR/G, and ∆G′ = 0.178 MPa for NR/CR/G-OTS), and therefore it is also the best dispersed in the vulcanizate. The addition of silane resulted in a reduction in the Payne effect and thus better dispersion of the filler. It is worth emphasizing that the addition a silane positively affected the biofiller’s dispersion in all NR/CR vulcanizates.

## 4. Conclusions

This paper describes the development of unconventional cross-linked and filled blends of natural and chloroprene rubbers (NR/CR). The paper proposes new eco-friendly, bio-based fillers for elastomeric materials. The results of the study demonstrated that ginseng, lemongrass, turmeric, or wood flour can be used as effective biofillers in elastomeric compositions, particularly blends containing natural rubber and chloroprene rubber (NR/CR).

The mechanical and dynamic properties of CR/NR vulcanizates are strongly dependent on the type of biofiller used and its possible silane modification. The vulcanizate containing ginseng demonstrated the best strength properties, including the highest tensile strength (TS_b_ = 7.22 MPa) and good resistance to thermo-oxidative aging (AF = 0.8). It was also characterized by the highest degree of cross-linking (α = 0.257). Wood flour as a filler provided high tear strength (T_s/max_ = 8.99 N/mm) and high hardness (HA = 59 °ShA). Although this material also generated the highest energy losses due to hysteresis, its advantages—including low price and easy availability as wood waste—make it particularly promising. Modification of fillers with silane most often led to increased cross-linking, tensile strength, stress, and hysteresis losses, while simultaneously reducing hardness and swelling. However, these effects were dependent on the specific biofiller. Silane modification can provide improved interfacial bonding with hydrophobic polymer matrices through increased compatibility, as silanization of biofillers allows for the attachment of functional groups that can bind to polymers. This modification can lead to improved mechanical properties, such as tensile strength and durability, in polymer composites.

Additional measurable benefits of using NR/CR compositions include eliminating the toxic vulcanization accelerators, shortening the cross-linking time to 5 min, and eliminating the undesirable reversion phenomenon.

In summary, the plant-derived fillers used are eco-friendly and significantly influence the properties of the final materials. Their selection should be tailored to the requirements of the specific application. Among the fillers tested, ginseng stands out for its greatest potential due to the favorable mechanical and economic properties of NR/CR vulcanizates filled with this filler type.

The next steps in the future research will focus on direct silanization of the proposed biofillers using various silanes. Another important issue will be the use of biofillers to fill other elastomeric matrices, based solely on natural rubber or its blends with epoxidized natural rubber.

## Figures and Tables

**Table 1:** Compositions of the NR/CR blends.

Ingredient	Ingredient’s Amount (phr)								
NR	50	50	50	50	50	50	50	50	50
CR	50	50	50	50	50	50	50	50	50
SA	1	1	1	1	1	1	1	1	1
ZnO	5	5	5	5	5	5	5	5	5
MgO	4	4	4	4	4	4	4	4	4
TM	0.5	0.5	0.5	0.5	0.5	0.5	0.5	0.5	0.5
CBS	1	1	1	1	1	1	1	1	1
S	2	2	2	2	2	2	2	2	2
G	-	30	30	-	-	-	-	-	-
LG	-	-	-	30	30	-	-	-	-
T	-	-	-	-	-	30	30	-	-
WF	-	-	-	-		-	-	30	30
OTS	-	-	2	-	2	-	2	-	2
Composite’s symbol	0	G	G-OTS	LG	LG-OTS	T	T-OTS	WF	WF-OTS

NR—natural rubber, CR—chloroprene rubber, SA—stearic acid, ZnO—zinc oxide, MgO—magnesium oxide, TM—thiourea, CBS—N-cyclohexylothiobenzosulfenamide, S—sulfur, G—ginseng, LG—lemongrass, T—turmeric, WF—wood flour, OTS—n-octadecyltrimethoxysilane, phr—parts per hundred of rubber.

**Table 2:** The cross-linking parameters of the unfilled or filled NR/CR (50/50 by wt.) blends.

Filler	Rheometric Parameters				
	t_02_ (min)	t_90_ (min)	T_min_ (dNm)	∆T_max_ (dNm)	CRI (min^−1^)
0	1.63	4.77	0.23	4.91	31.85
G	1.33	5.18	0.24	6.63	25.97
G-OTS	1.34	4.97	0.29	5.53	27.55
LG	1.31	4.69	0.24	5.96	29.59
LG-OTS	1.14	5.78	0.27	5.64	21.55
T	1.53	3.69	0.24	4.07	46.30
T-OTS	1.25	6.40	0.28	5.58	19.42
WF	1.53	5.27	0.25	6.33	26.74
WF-OTS	1.29	5.71	0.29	6.06	22.62

T_min_—minimum torque, ∆T_max_—maximum torque increment, t_02_—scorch time, t_90_—cure time, CRI—cure rate index.

**Table 3:** Differential scanning calorimetry (DSC) results from unfilled or filled NR/CR (50/50 by wt.) blends.

Symbol	T_g NR_	T_g CR_	T_onset_	T_endset_	ΔH
	°C	°C	°C	°C	J/g
0	−62.7	−38.4	175	221	16.93
G	−62.8	−38.6	157	219	32.92
G-OTS	−62.8	−38.7	168	225	26.13
LG	−62.1	−37.5	170	221	23.53
LG-OTS	−62.6	−38.2	166	221	26.71
T	−62.1	−38.6	133	203	23.08
T-OTS	−62.3	−38.2	132	223	26.72
WF	−61.9	−38.4	170	220	26.43
WF-OTS	−61.8	−37.7	179	221	25.45

T_g NR_—glass-transition temperature of NR; T_g CR_—glass-transition temperature of CR; T_onset_—the onset temperature of the cross-linking; T_endset_—the endset temperature of the cross-linking; ∆H—enthalpy of the cross-linking.

**Table 4:** Results of the equilibrium swelling test of the unfilled or filled NR/CR (50/50 by wt.) vulcanizates.

Filler	Swelling Parameters					
	In Toluene			In Hexane		
	Q_v_^T^ (mL/mL)	W_Q_^T^ (mg/mg)	V_r_^T^	Q_v_^H^ (mL/mL)	W_Q_^H^ (mg/mg)	V_r_^H^
0	4.48 ± 0.09	0.06 ± 0.01	0.183 ± 0.001	1.29 ± 0.07	0.04 ± 0.01	0.437 ± 0.001
G	3.89 ± 0.05	0.05 ± 0.01	0.205 ± 0.002	1.29 ± 0.07	0.04 ± 0.01	0.436 ± 0.001
G-OTS	4.00 ± 0.10	0.06 ± 0.01	0.200 ± 0.001	1.22 ± 0.03	0.05 ± 0.01	0.450 ± 0.001
LG	4.30 ± 0.02	0.05 ± 0.01	0.189 ± 0.001	1.29 ± 0.04	0.05 ± 0.01	0.448 ± 0.002
LG-OTS	4.17 ± 0.06	0.06 ± 0.01	0.190 ± 0.003	1.23 ± 0.04	0.06 ± 0.01	0.449 ± 0.003
T	5.78 ± 0.15	0.05 ± 0.01	0.148 ± 0.001	1.62 ± 0.04	0.05 ± 0.01	0.381 ± 0.001
T-OTS	4.61 ± 0.04	0.06 ± 0.01	0.178 ± 0.001	1.33 ± 0.07	0.05 ± 0.01	0.429 ± 0.002
WF	4.60 ± 0.15	0.05 ± 0.01	0.178 ± 0.002	1.33 ± 0.12	0.05 ± 0.01	0.430 ± 0.002
WF-OTS	4.41 ± 0.07	0.06 ± 0.01	0.185 ± 0.002	1.14 ± 0.02	0.06 ± 0.01	0.468 ± 0.001

Q_v_^T^—equilibrium volume swelling degree in toluene, W_Q_^T^—the content of the eluted fraction in toluene, V_r_^T^—rubber volume fraction in toluene, Q_v_^H^—equilibrium volume swelling degree in hexane, W_Q_^H^—the content of the eluted fraction in hexane, V_r_^H^—rubber volume fraction in hexane.

**Table 5:** The results of the strength properties of unfilled and filled NR/CR (50/50 by wt.) vulcanizates before and after thermo-oxidative aging (70 °C, 7 days).

Properties	Filler								
	0	G	G-OTS	LG	LG-OTS	T	T-OTS	WF	WF-OTS
**Before thermo-oxidative aging**									
S_e100_ (MPa)	0.79 ± 0.03	1.23 ± 0.02	1.20 ± 0.06	1.36 ± 0.04	1.46 ± 0.06	0.71 ± 0.03	0.95 ± 0.05	1.40 ± 0.04	1.66 ± 0.25
S_e200_ (MPa)	1.20 ± 0.06	1.74 ± 0.03	1.67 ± 0.06	1.74 ± 0.05	1.85 ± 0.05	0.96 ± 0.02	1.27 ± 0.03	1.77 ± 0.04	2.07 ± 0.12
S_e300_ (MPa)	1.60 ± 0.09	2.13 ± 0.03	2.05 ± 0.08	2.10 ± 0.06	2.20 ± 0.06	1.17 ± 0.02	1.58 ± 0.04	2.09 ± 0.05	2.41 ± 0.15
TS_b_ (MPa)	6.31 ± 0.30	7.22 ± 0.05	6.93 ± 0.14	6.66 ± 0.24	6.91 ± 0.34	3.94 ± 0.18	5.53 ± 0.23	6.29 ± 0.17	6.90 ± 0.38
E_b_ (%)	994 ± 10	990 ± 8	993 ± 15	993 ± 11	995 ± 6	991 ± 17	996 ± 12	992 ± 20	970 ± 13
After thermo-oxidative aging (70 °C, 7 days)									
S_e100_* (MPa)	0.83 ± 0.03	1.28 ± 0.04	1.26 ± 0.03	1.50 ± 0.03	1.49 ± 0.06	0.86 ± 0.01	1.16 ± 0.02	1.73 ± 0.04	1.82 ± 0.10
S_e200_* (MPa)	1.30 ± 0.04	1.75 ± 0.05	1.70 ± 0.03	1.82 ± 0.05	1.79 ± 0.06	1.17 ± 0.01	1.51 ± 0.04	2.03 ± 0.04	2.08 ± 0.07
S_e300_* (MPa)	1.75 ± 0.05	2.13 ± 0.07	2.05 ± 0.05	2.17 ± 0.07	2.16 ± 0.08	1.45 ± 0.01	1.89 ± 0.04	2.36 ± 0.06	2.43 ± 0.05
TS_b_* (MPa)	6.70 ± 0.27	6.60 ± 0.08	6.39 ± 0.30	5.98 ± 0.52	6.05 ± 0.57	4.56 ± 0.18	6.16 ± 0.23	5.53 ± 0.83	5.17 ± 0.19
E_b_* (%)	870 ± 21	865 ± 32	924 ± 10	886 ± 41	891 ± 38	925 ± 10	924 ± 15	834 ± 52	684 ± 27

S_e100_, S_e200_, S_e300_—stress at an elongation of 100%, 200%, or 300%; TS_b_—tensile strength; E_b_—elongation at break; S_e100_*, S_e200_*, S_e300_*—stress at an elongation of 100%, 200%, or 300% after thermo-oxidative aging; TS_b_*—tensile strength after thermo-oxidative aging; E_b_*—elongation at break after thermo-oxidative aging.

**Table 6:** Results of the tear resistance of unfilled and filled NR/CR (50/50 by wt.) vulcanizates.

Filler	Tear Strength		
	Measuring Path (mm)	T_s/med_ (N/mm)	T_s/max_ (N/mm)
0	62	3.75 ± 0.33	5.47 ± 0.48
G	206	5.64 ± 0.34	6.49 ± 0.62
G-OTS	155	5.15 ± 0.14	6.03 ± 0.67
LG	83	6.34 ± 0.63	8.21 ± 0.92
LG-OTS	78	5.16 ± 0.15	6.42 ± 0.06
T	79	3.51 ± 0.17	4.18 ± 0.27
T-OTS	87	4.88 ± 0.63	6.16 ± 0.83
WF	127	7.43 ± 0.03	8.99 ± 0.04
WF-OTS	136	10.8 ± 0.20	12.90 ± 0.56

T_s/med_–average tearing force, T_s/max_–maximum tearing force.

**Table 7:** Results of the hysteresis of unfilled and filler NR/CR (50/50 by wt.) vulcanizates.

Filler	Hysteresis	
	∆W_i_ (Nmm)	E_M_ (%)
0	34.1 ± 0.4	10.5 ± 0.6
G	105.5 ± 1.9	38.9 ± 0.1
G-OTS	106.4 ± 4.6	41.7 ± 3.5
LG	145.0 ± 16.2	52.3 ± 2.0
LG-OTS	132.6 ± 0.38	57.9 ± 1.5
T	77.2 ± 4.8	38.2 ± 1.3
T-OTS	148.7 ± 5.5	52.7 ± 3.2
WF	164.7 ± 0.6	52.8 ± 1.5
WF-OTS	191.5 ± 12.1	57.0 ± 1.3

∆W_i_—hysteresis losses, E_M_—Mullins effect.

**Table 8:** Results of the dynamic properties of unfilled and filler NR/CR (50/50 by wt.) vulcanizates.

Filler	G′_max_ (MPa)	G″_max_ (MPa)	∆G′ (MPa)
0	0.165	0.053	0.151
G	0.308	0.049	0.300
G-OTS	0.212	0.031	0.178
LG	0.395	0.076	0.344
LG-OTS	0.245	0.041	0.188
T	0.783	0.152	0.732
T-OTS	0.521	0.092	0.475
WF	0.779	0.164	0.677
WF-OTS	0.713	0.111	0.640

G′_max_—maximum storage modulus, G″_max_—maximum loss modulus, ∆G′—Payne effect.

## Data Availability

The original contributions presented in this study are included in the article. Further inquiries can be directed to the corresponding author.
